# Neuropsychological Impairment in Detoxified Alcohol-Dependent Subjects with Preserved Psychosocial Functioning

**DOI:** 10.3389/fpsyt.2017.00193

**Published:** 2017-09-29

**Authors:** Catherine Martelli, Amélie Petillion, Marine Brunet-Lecomte, Rubén Miranda Marcos, Sandra Chanraud, Ammar Amirouche, Alexia Letierce, Nikoleta Kostogianni, Hervé Lemaitre, Henri-Jean Aubin, Lisa Blecha, Michel Reynaud, Jean-Luc Martinot, Amine Benyamina

**Affiliations:** ^1^AP-HP, Paul Brousse Hospital, Department of Psychiatry and Addictology, Villejuif, France; ^2^INSERM U1018, Centre de recherche en Epidémiologie et Santé des Populations, Villejuif, France; ^3^Université Paris-Sud, Le Kremlin Bicêtre, France; ^4^INSERM, U1000 “Neuroimaging & Psychiatry”, IFR49, Orsay, France; ^5^CEA, “Neuroimaging & Psychiatry” U1000 Unit, Hospital Department Frédéric Joliot, Orsay, France; ^6^Bordeaux University, INCIA, UMR 5287, Talence, France; ^7^CNRS, INCIA, UMR 5287, Talence, France; ^8^AP-HP, Bicêtre Hospital, Clinical Research Unit, Le Kremlin Bicêtre, France; ^9^Université Paris-Sud, Orsay, France; ^10^Université Paris Descartes, UMR U797, Paris, France

**Keywords:** alcohol use disorder, alcohol dependence, executive functions, episodic memory, working memory

## Abstract

**Background:**

Chronic alcoholism and its related cognitive impairments are associated with increased social, relational, and professional deficits which have a variable overall impact on social integration. These impairments are known to have varying severities and have rarely been studied among healthy alcohol-dependent subjects with preserved psychosocial functioning. Thus, the objective of this study is to describe neuropsychological performance in this particular population.

**Method:**

Twenty-nine socially adjusted alcohol-dependent men, hospitalized for a first or second withdrawal and abstinent for 3 weeks minimum, were compared to 29 healthy non-alcoholic controls. All subjects underwent clinical and psychiatric examination, neuropsychological tests of memory (M), working memory (WM), and executive functions (EF). Comparisons were performed using Student’s *t*-tests or Mann–Whitney *U* tests.

**Results:**

No group differences were found on the Self-Reported Social Adjustment Scale (SAS-SR) or in the Mini-Mental State Examination. Compared to controls, patients had greater episodic, spatial, and WM deficits as well as slightly altered executive functions. In contrast, their executive functions (spontaneous flexibility, criteria generation, rule maintenance, and inhibitory control) were relatively preserved.

**Conclusion:**

Our sample of socially and professionally integrated alcoholic patients shows fewer cognitive deficits than described in previous studies. Our results suggest that early on, alcohol-dependent subjects develop compensatory adaptation processes to preserve social function and adaptation. Minor cognitive impairments should be screened early in the disease to integrate cognitive interventions into the health-care plan to thus eventually prevent further socio-professional marginalization.

## Introduction

Fifty-five million adults drink at harmful levels in the EU. An estimated 23 million of them have alcohol use disorder (1). It is associated with cognitive modifications such as slower processing speeds (2, 3), impaired episodic memory (4–8), lesser visual–spatial skills (9–11), reduced working memory (WM) (12–17), and attention (18). Executive functions including planning (19), decision making (20), cognitive flexibility (21), problem solving (22), and inhibition (23–25) may also be impaired.

A previous study by Chanraud et al. (26) investigated the relationship between regional alterations, drinking history, and executive performance among detoxified alcohol dependents with preserved psychosocial functioning. The results showed a correlation between neuropsychological alterations and decreased gray and white matter volumes.

Job performance in alcoholic patients may be linked with cognitive status. Studies by Donovan et al. (27) and Walker et al. (28) have shown a parallel between cognitive impairment and employment status in alcoholic patients. Posttreatment employment performance appears to be predicted by baseline professional integration. Alcohol-dependent subjects who worked full time had higher scores at 6-month follow-up on neuropsychological measures than those who were unemployed or worked part-time. Baseline neuropsychological status was found to be a statistically significant predictor of employment success 9 months after discharge from treatment (27).

Alcohol-dependent subjects suffer from impaired social cognition, which contributes to interpersonal problems, discomfort, and stress in social situations. However, to the best of our knowledge, social adjustment is rarely formally measured nor reported. One exception is a study by Fein et al. (29) showing that patients with long-term abstinence, who seemed to maintain proper social integration, were relatively free of neuropsychological impairment except for minor deficits in spatial processing. In addition, supportive social networks would seem to improve substance use outcomes particularly in alcohol-dependent subjects with executive or memory impairments (30, 31).

The objective of this study is to examine overall neuropsychological functioning in clinically healthy detoxified alcohol-dependent (DAD) patients who remain socially integrated [social functioning was evaluated using the self-reported social adjustment scale (SAS-SR)], using a series of tests evaluating three cognitive domains: memory (M), WM, and executive functions (EF) versus non-alcoholic controls. DAD patients and controls were all full-time employed.

Our hypothesis is that DAD patients will show preserved executive functions and memory thus enabling them to properly function in their socio-professional environment.

## Materials and Methods

### Participants

Twenty-nine Caucasian DAD men (25–65 years old) were recruited on admission for withdrawal or day-hospital units in the addiction departments of Paul Brousse and Emile Roux Hospital (Assistance Publique, Hopitaux de Paris). Thirty-one patients had previously participated in a study correlating cerebral volumetry and neuropsychological data (26). A senior psychiatrist interviewed patients, performed a clinical examination and reviewed patient’s medical records and biological data. IQ was not evaluated. Sociodemographic and clinical data are presented in Tables 1 and 2 (drinking history variables).

**Table 1:** Characteristics of participants.

	DAD (*n* = 29)	Controls (*n* = 29)	*p*-Value
Age (years)	47.41 ± 7.68	45.06 ± 8.43	0.27^†^
Education (years)	11.62 ± 3.27	13.03 ± 3.44	0.12^†^
BMI	24.14 ± 3.84	24.75 ± 3.42	0.57^†^
SAS-SR	2.44 ± 0.52	2.57 ± 0.52	0.36^†^
MMSE	29.03 ± 2.00	29.31 ± 1.10	0.72^†^
Tobacco consumption	72.4%	13.8%	<0.0001^‡^
Fagerström (FTND)	3.85 ± 3.34	0.89 ± 2.02	<0.001
Years of tobacco consumption	19.92 ± 13.30	3.48 ± 8.88	<0.0001^†^
Cigarettes per day	18.27 ± 13.92	2.60 ± 6.31	<0.0001^†^
Pack-years	25.27 ± 21.13	2.92 ± 8.18	<0.0001^†^

*M, mean, range: minimum–maximum*.

*BMI, body mass index; SAS-SR, Self-reported social adjustment scale; MMSE, Mini-Mental State Examination, pack-years (number of cigarettes per day/20 × years of tobacco consumption); FTND, Fagerström Test of Nicotine Dependence; DAD, detoxified alcohol-dependent*.

*^†^t-test*.

*^‡^Chi^2^ test*.

**Table 2:** Drinking history variables.

	Alcoholics (*n* = 29)	
	Mean ± SD	Range
Age of first drink (years)	16 ± 2.5	10–20
Age of alcohol dependence onset (years)	38.06 ± 10.34	20–56
Total years of alcohol dependence	8.15 ± 7.25	1–29
Quantity of alcohol used^a^ (UIA)	30.48 ± 16.89	7–66
Number of prior withdrawals	0.86 ± 0.78	0–2
Abstinence duration^b^ (weeks)	50.79 ± 53.79	3–182
Ethanol biomarkers (laboratory norms)		
CDT (<2.6%)^b^	1.97 ± 0.25	
GGT (<53)^b^	41.65 ± 35.18	10–110
AST (<38 U/L)^b^	27.88 ± 14.11	16–42
ALT (<40 U/L)^b^	26.92 ± 14.93	14–70
MCV (80–100 fL)^b^	94.08 ± 6.59	80.8–110
Bilirubin (12–17 g/dL)^b^	12.00 ± 4.37	3–20
Hemoglobin (13–16.5 g/dL)^b^	14.94 ± 1.11	12.1–16.6
Hematocrit (40–54%)^b^	44.05 ± 3.30	36.2–47.8
Rating scales		
BDI-13	3.88 ± 3.21	0–10
HDRS	1.70 ± 1.35	0–5
HARS	1.88 ± 1.36	0–5
AUDIT	33.11 ± 4.33	23–40

*^a^Drinks per day over the 3-month preceding detoxification. Drinks of each type of alcoholic beverage (wine, beer, and spirit) were standardized to units containing approximately 10 g of absolute alcohol (UIA)*.

*^b^Laboratory norms*.

*CDT, carbohydrate-deficient transferrin; GGT, gamma-glutamyl-transferase; AST, aspartate aminotransferase; ALT, alanine aminotransferase; MCV, mean corpuscular volume; BDI-13, Beck Depression Inventory short form; HDRS, Hamilton Depression Rating Scale; HARS, Hamilton Anxiety Rating Scale; AUDIT, Alcohol Use Disorders Identification Test*.

All patients met DSM IV diagnostic criteria for alcohol dependence (32). All patients had a history of maximum two prior hospitalizations for withdrawal to minimize potential cognitive deficits induced by supplementary withdrawals (33). All patients had been abstinent for at least 3 weeks as shown by normalized gamma-glutamyl-transferase (GGT), mean corpuscular volume (MCV), and carbohydrate-deficient transferrin (CDT) levels (see Table 2). During withdrawal, a standard treatment protocol (vitamin B1 and B6 and digressive diazepam) was used. At the time of testing, all sedation had ceased for at least 7 days. Nine patients identified at least one first degree relative with a drinking problem. Even though four patients reported lifetime maternal drinking, none reported any maternal alcohol use during pregnancy.

Control subjects who drank less than two standard units of alcohol per week (20 g) during the previous year and had a score of ≤5 on the Alcohol Use Disorders Identification Test (AUDIT) (34) were recruited from the neighboring community (*n* = 29). Controls were compensated (100 Euros) by the study’s sponsor (INSERM).

Patients and controls were excluded, after a clinical interview with a senior psychiatrist, according to DSM-IV criteria, if they had a prior history of substance dependence or abuse (except tobacco and caffeine), axis I disorder (particularly mood and/or anxiety disorders, psychosis), high scores on Hamilton anxiety and Hamilton depression scales (>5), any current or past disease which could impair cognitive functions: hepatic (cirrhosis, hepatitis), neurological (seizures, encephalopathy, Wernicke–Korsakoff syndrome), cardiovascular, renal, or head trauma (loss of consciousness for more than 30 min), stroke, signs or symptoms of malnutrition or major brain abnormalities on MRI scan. Axis II was not considered as an exclusion criterion. Two participants were excluded: one because of a microinfarctus in the perithalamic region on MRI scan; and another because he chose not to complete the neuropsychological testing. None refused to participate. Our final analysis included 29 alcohol-dependent subjects and 29 male controls.

All participants received verbal and written protocol information and signed a consent form prior to inclusion. This protocol was approved by the Bicêtre Hospital ethics committee.

### Laboratory Assessments

On the day of testing, fasting blood samples were taken to investigate the somatic complications of chronic alcoholism. The panel of tests included liver function tests: albumin, alkaline phosphatase, bilirubin, aspartate aminotransferase (AST), alanine aminotransferase (ALT), GGT, and CDT. In addition, blood chemistry (complete blood count, MCV, mean corpuscular hemoglobin concentration, hemoglobin, hematocrit, serum protein, and serum sodium levels) was assessed (see Table 2).

### Rating Scales

After eating, subjects were evaluated using the AUDIT to assess the degree of alcohol dependence. Social functioning was evaluated using the Self-reported social adjustment scale (SAS-SR) (35), a self-administered questionnaire including questions on social and leisure activities, relationships with the extended family, marital partner, offspring, and relationships within the family circle. It also provides information concerning socio-economic status. Each item is scored on a 5-point scale with higher scores indicating poorer functioning. The total SAS-SR score is calculated by averaging all items.

Nicotine dependence was evaluated using the Fagerström Test of Nicotine Dependance (FTND) (36) measuring cigarettes smoked per day, pack-years, and smoking duration. Pack-years were calculated as [(number of cigarette per day/20) × (duration of smoking in years)].

Body mass index (BMI) was calculated by dividing weight (kg) by height squared (m^2^) providing a good instrument to control health risk.

### Neuropsychological Assessment

All participants underwent the Mini-Mental State Examination (MMSE) and a battery of neuropsychological tests conducted by a senior neuropsychologist, according to standardized procedures. Subjects were tested individually in a single session with a standardized test order.

The battery of neuropsychological tests assessed three cognitive domains and measured memory (M), WM, and executive functions (EF). The tests are briefly described below and detailed in the Supplementary Material.

Episodic memory was assessed by the Rey–Osterrieth Complex Figure (ROCF), Free and Cued Selective Reminding Test. The information subtest of the Wechsler Adult Intelligence Third Revision (WAIS III) was used to assess semantic memory.

Working memory was assessed by Digit Span subtest, Spatial Span subtest, and Letter-number sequencing subtest (WAIS III).

We used the Trail Making Test part A and B (TMT A/B), Wisconsin Card Sorting Test (WCST), Stroop test, and Letter Fluency test to evaluate executive functions.

All tests were scored according to standard published procedures (see Supplementary Material).

### Statistical Analyses

Statistical analyses were conducted using R software (http://www.R-project.org/). The normality of variable distribution was assessed by the Shapiro–Wilk test. Two-sided comparisons between groups were made for age, education, BMI, SAS-R, and tobacco consumption variables using either Student’s *t*-test for normally distributed values or Mann–Whitney *U* test for non-normally distributed values, with a level of 0.05 or less. Then, *Z*-scores for the psychological tests were calculated to compare alcoholic patients with controls, adjusted by years of education. The mean and SD of each test was calculated for the control group, donated *m* and *s*, and the *z*-score for an alcoholic subject was defined as *z* = (score − *m*)/*s*.

## Results

Results on the neuropsychological assessment are presented in Table 3.

**Table 3:** Comparison of neuropsychological testing between detoxified alcohol-dependent patients and controls.

	DAD (*n* = 29)	Controls (*n* = 29)	*t*(56)/*U*	
	Mean ± SD	Mean ± SD	*p*-Value	Adjusted *p*-value
MMSE	29.03 ± 2.01	29.31 ± 1.11	0.822	0.797
**ROCF**				
Copy	33.17 ± 2.69	35.76 ± 0.69	**<0.00001**	**<0.00001**
Recall copy	14.62 ± 6.81	23.62 ± 6.28	**<0.0001**	**<0.0001**
**FCSRT**				
Immediate cued recall	15.28 ± 1.07	14.59 ± 1.62	0.076	0.059
Free recall	28.83 ± 7.22	38.07 ± 5.37	**<0.00001**	**<0.00001**
Total recall	44.66 ± 3.15	47.07 ± 1.69	**<0.0003**	**0.0002**
Free delayed recall	11.93 ± 2.59	14.59 ± 1.57	**<0.00001**	**<0.0001**
Total delayed recall	15.65 ± 0.67	15.79 ± 0.77	0.283	0.115
Recognition	15.89 ± 0.56	15.90 ± 0.41	0.840	0.598
**WAIS III**				
Information	8.31 ± 3.63	11.76 ± 3.43	**0.0017**	**0.0016**
**WAIS III**				
Digit span total	9.3 ± 3.00	11.31 ± 2.66	**0.0105**	**0.0098**
Digit span forward	6.03 ± 1.59	6.62 ± 1.05	0.0886	0.0803
Digit span backward	4.10 ± 1.47	5.31 ± 1.17	**0.0026**	**0.0019**
Letter-number sequencing	8.31 ± 3.78	11.28 ± 2.93	**0.001**	**0.006**
**WMS-III**				
Spatial span	7.00 ± 3.41	11.21 ± 2.35	**<0.001**	**<0.001**
TMT-A (s)	40.80 ± 11.90	33.00 ± 11.90	**0.0092**	**0.0092**
TMT-B (s)	125.40 ± 66.80	82.60 ± 41.30	**0.0041**	**0.0041**
**WCST**				
Categories completed	5.07 ± 1.79	5.93 ± 0.37	**0.009**	**0.035**
Number of errors	27.40 ± 32.04	18.00 ± 12.12	0.2985	0.2982
Number of perseverative errors	20.27 ± 26.20	11.07 ± 9.34	0.0751	0.0747
**Stroop test**				
Word reading	42.69 ± 9.68	46.45 ± 6.17	0.084	0.140
Color naming	43.10 ± 11.60	48.31 ± 9.20	0.063	0.125
Color word reading	44.40 ± 11.30	49.00 ± 9.77	0.104	0.160
Interference	49.34 ± 8.44	53.10 ± 11.10	0.158	0.573
**Verbal fluency**				
Category	25.41 ± 5.59	25.52 ± 6.06	0.946	0.818
Letter fluency P	18.90 ± 5.52	21.76 ± 5.46	0.052	0.111
Letter fluency R	17.28 ± 5.65	18.55 ± 4.84	0.614	0.582

*MMSE, Mini-Mental State Examination; FCSRT, Free and Cued Selective Reminding test; ROCF, Rey–Osterrieth Complex Figure; WCST, Wisconsin Card Sorting Test; TMT, Trail Making Test; WMS-III, Wechsler Memory Scale Third Edition; WAIS III, Wechsler Adult Intelligence Third Revision; DAD, detoxified alcohol-dependent*.

*p Values for between-group comparisons with Mann–Whitney U tests. Adjusted p-value: p-value for the group effect, adjusted for the years of education. Bold p-values indicate a significant difference between groups (p < 0.05)*.

### Population Characteristics

Our final analysis included 29 DAD men and 29 male controls. The DAD and control groups were comparable in terms of age, years of education, BMI, SAS-SR, and MMSE (Table 1). The patients had greater tobacco consumption than controls as shown by FTND. The DAD group showed a significantly greater dependence score versus the control group (respectively, 3.85 ± 3.34 versus 0.89 ± 2.02; *p* < 0.001). DAD tobacco consumption was significantly greater than in the control group as shown by years of tobacco use (*p* < 0.0001) and the number of cigarettes per day (*p* < 0.0001).

All subjects were employed full-time and were in a stable relationship. Patients were either married (*n* = 20) or cohabiting (*n* = 9). All had a fixed address and lived in a house or flat. Our patients belonged to various socio-professional groups: artisans, small business owners (*n* = 7), teachers and scientific professions (*n* = 9), administrative and trained personnel (*n* = 7), and blue collar workers (*n* = 6).

All patients had undergone a maximum of two prior hospitalizations for withdrawal. Among our final patient sample, 7 had undergone two prior alcohol detoxifications, while the other 22 had only one prior detoxification. All patients had been abstinent for at least 3 weeks as shown by normalized GGT, MCV, and CDT levels (see Table 2).

### Executive Functions (EF)

Detoxified alcohol-dependent participants have less efficient executive functioning. Compared to controls, DAD patients had significant impairments on set-shifting and reactive mental flexibility (TMT B, *p* = 0.0041), and a significant difference was found between the two groups on criteria generation (WCST, *p* = 0.035). We also observed significant impairments in planning ability and organization as assessed by the figure copy (ROCF, *p* < 0.00001). No significant differences were found between the two groups in performance on inhibitory control (Stroop), spontaneous mental flexibility (Letter fluency), and perseverative errors (WCST) (see Table 3).

### Memory (M)

No differences were found for patients compared to controls for immediate cued recall, total delayed recall and recognition in the verbal episodic memory test. However, DAD subjects had lower scores than controls in free recall (*p* < 0.00001), total recall (*p* = 0.0002), and free delayed recall (*p* < 0.0001).

Visual episodic memory capacities were impaired in DAD patients group versus controls group (ROCF recall copy, *p* < 0.0001). Semantic memory score was also lower in DAD subjects compared to controls (WAIS III, information subtest, *p* = 0.0016) (see Table 3).

### Working Memory

Detoxified alcohol-dependent subjects presented alterations in most WM tasks. Forward digit span was not significantly altered (*p* = 0.0803), whereas backward digit span was significantly diminished (*p* = 0.0019) (WAIS III). In addition, DAD alcoholic subjects performed significantly worse than controls on letter-number sequencing (*p* = 0.006). Spatial span was also diminished (Wechsler Memory Scale Third Edition, *p* < 0.001) (see Table 3).

## Discussion

Our study confirms that there are neurocognitive deficits in detoxified alcoholic subjects. As described in several studies, alcoholic patients exhibit neuropsychogical deficits, in particular executive impairments and impaired memory abilities, partially caused by structural damage to frontocerebellar and Papez’s circuits as well as functional modifications (26, 37).

The objective of this study is to describe neuropsychological performances in DAD subjects, with preserved psychosocial functioning, compared to controls.

### Executive Functions

Many studies have described a dysexecutive syndrome in alcoholic patients (20, 23, 25, 38–40). Our results showed reactive cognitive flexibility impairments and visual and verbal memory retrieval deficits. Similar to Sullivan et al. (11), we observed impairment in planning ability and organization as assessed by the ROCF copy. DAD patients used a copying strategy with a “bits and pieces” approach rather than a global structured approach. These results are consistent with a previous study suggesting that alcoholic patients use less efficient strategies to perform visuoperceptual learning tasks (41). Those impairments persist even after 6 months abstinence, as described by Munro et al. (10).

Despite these mild cognitive impairments, other cognitive functions would seem to be preserved in our DAD participants. Indeed, inhibitory control can allow them to resist acting impulsively, and more specifically, control and curb alcohol cravings. Moreover, self-control is having the discipline to stay focused and to complete a task, for example, at work.

By having preserved spontaneous flexibility abilities, DAD participants seem to be able to adapt their relationship, adjust their behavior in interaction and respond to unanticipated situations despite reactive cognitive flexibility impairment.

In addition, being able to maintain rules without any perseverative tendency, as controls, DAD patients are enable to plan for a solution by selecting efficient strategies, to sustain goals or instructions, and to dynamically adjust their response to a feedback from the environment.

Thus, our results show that alcohol-dependent patients who have maintained a professional integration show partially preserved executive functions that enable them to stay effective in their work and adapted in social interactions.

### Memory

Our results show episodic memory deficits for visual material and spatial processing among DAD subjects, compared to controls. More precisely, our results suggest that consolidation or retention are preserved but retrieval is impaired (total recall and free delayed recall deficits associated with normal cued delayed recall). Other authors have also shown that alcohol-dependent subjects presented lower performance than controls in verbal episodic memory (19, 42, 43).

These memory deficits may be partially explained by executive dysfunction. Many authors have shown that executive functions are involved in encoding and retrieval (25, 44). Other authors have hypothesized that this may be due to inefficient retrieval strategies in alcohol-dependent subjects and not to storage capacities (45, 46).

Semantic memory and verbal knowledge tested by the information subtest of the WAIS III is significantly altered in DAD patients. Pitel et al. (44) showed that alcohol-dependent subjects presented impaired novel semantic learning (shallower encoding) and that alcohol-dependent patients used inefficient cognitive strategies to compensate for cognitive deficits.

Detoxified alcohol-dependent subjects presented significant impairments in most WM tasks. The deficits in forward digit span or spatial span, and in more complex information processing such as backward restitution and letter-number sequencing, suggest that alcohol-dependent patients have a limited ability to manipulate information due to impaired WM. Noel et al. (23) observed a similar result on the alpha-span task.

Working memory involves systems implicated in short-term maintenance and manipulation of information necessary to accomplish complex tasks. Baddeley’s model included two slave systems to ensure temporary information maintenance, the phonological loop and the visuo-spatial sketchpad, and a central executive system considered to be similar to the executive functions (47).

Thus, our subjects seem to present deficits in key systems at all levels of Baddeley’s model even though they maintain preserved psychosocial functioning. These findings suggest that alcohol-dependent subjects developed compensatory mechanisms, so they can preserve socio-professional adaptation.

### Limitations and Directions for Future Research

All subjects recruited in this study are men. Thus, it is impossible to generalize our results to all alcoholic subjects. This study evaluated specific controls with particularly low alcohol consumption levels. Thus, the results from our controls may not reflect results in the general population. Among DAD patients, we can notice a wide variability length of abstinence (from 3 weeks to 3.5 years) that could influence the results.

This could also be explained by the relatively few detoxifications in this sample of patients. It has been shown that the number of reported alcohol withdrawals during the prior year was a significant predictor of poorer cognitive performance following detoxification (48, 49).

Another important question is whether the improvement of certain memory and executive cognitive functions would predict greater motivation to change drinking?

To answer these questions, it would be interesting to prospectively investigate this population following long-term detoxification with an evaluation of drinking behavior change, relapse, employment, and the social adjustment outcomes.

There was also a significant difference in tobacco consumption between the DAD population and the control group in this study. The literature often shows a strong correlation between the consumption of alcohol and tobacco in alcohol-dependent subjects (50). However, we did control for this variable in our analyses, and thus our results on neuropsychological variables were independent of this difference. Other studies have shown that continued tobacco use may have a significant impact on cognitive function as well as future dementia (51, 52). Further studies should examine an eventual synergistic role between continued alcohol and tobacco use in cognitive decline in DAD patients, as well as the potential impact withdrawal.

We have interpreted the cognitive functioning alteration as resulting from alcohol abuse. An alternative explanation is that neuropsychological deficits may precede alcohol abuse and represent vulnerability or predisposition to alcohol dependence. The question of whether cognitive deficits in alcoholic patients are a consequence or a predisposition to the disease is still a matter of controversy and this could be the object of other prospective studies.

## Conclusion

Detoxified alcohol-dependent patients present WM, episodic and spatial memory deficits, and partially executive impairments compared to controls. However, they present preserved executive functions such as inhibitory control, spontaneous flexibility, and generation and rules maintaining.

Their preserved executive functions and the development of compensatory strategies may enable them to maintain socio-professional integration. Indeed, the results show their abilities to identify high-risk situations, to use appropriate coping skills in a given situation, to change perspectives, and adjust to new demands or priorities at work.

To prevent executive and memory deficits from developing, it seems necessary to screen for these cognitive deficits early on. This could enable the care team to offer cognitive remediation, to exercise cognitive functions, as we know that cognitive deficits in alcoholism can increase loss of control, contribute to further progression of the disease and increase relapse rates. Cognitive remediation could therefore help alcohol-dependent patients maintaining abstinence and socio-professional integration.

## Ethics Statement

All participants received verbal and written protocol information and signed a consent form prior to inclusion. This protocol was approved by the Bicêtre Hospital ethics committee.

## Author Contributions

CM is the primary investigator and was responsible for designing this study; responsible for the context and discussion writing. AP implicated in the analysis and selection of the neuropsychological tests for this study, as well as refining the results section. MB-L implicated in the analysis and selection of the neuropsychological tests for this study, as well as refining the results section. RMM implicated in the analysis and selection of the neuropsychological tests for this study and discussion of the results. SC participated in the study design and the reflection concerning the imaging analysis and correlations with clinical observations. AA participated in the methodological validation and statistical analysis of the data. AL participated in the methodological validation and initial statistical analysis of the data. NK participated in patients’ neuropsychological testing and test choice. HL participated in passage and reflection of neuroimaging protocol for this study. H-JA participated in recruiting patients, manuscript revision. LB participated and coordinated the writing, reflection (context, discussion, conclusions), and revision of the article. MR participated in the initial protocol design, conception and feasibility of this study in alcoholic patients; revision and reflection around the different versions of the manuscript. J-LM participated in the neuroimaging protocol design, feasibility; participation in manuscript revision. AB participated in the conception, reflection, and design.

## Conflict of Interest Statement

None of the authors has any conflicts of interest to declare regarding this study and its results.

## References

[1] World Health Organization. Global Status Report on Alcohol and Health 2014. Geneva: World Health Organization (2014).

[2] Stavro K Pelletier J Potvin S. Widespread and sustained cognitive deficits in alcoholism: a meta-analysis. Addict Biol (2013) 18:203–13. 10.1111/j.1369-1600.2011.00418.x 22264351

[3] Tedstone D Coyle K. Cognitive impairments in sober alcoholics: performance on selective and divided attention tasks. Drug Alcohol Depend (2004) 75:277–86. 10.1016/j.drugalcdep.2004.03.005 15283949

[4] Bernardin F Maheut-Bosser A Paille F. Cognitive impairments in alcohol-dependent subjects. Front Psychiatry (2014) 5:78. 10.3389/fpsyt.2014.00078 25076914 PMC4099962

[5] Cabe N Laniepce A Ritz L Lannuzel C Boudehent C Vabret F [Cognitive impairments in alcohol dependence: from screening to treatment improvements]. Encephale (2016) 42:74–81. 10.1016/j.encep.2015.12.012 26774623

[6] Noel X Van der Linden M Brevers D Campanella S Hanak C Kornreich C The contribution of executive functions deficits to impaired episodic memory in individuals with alcoholism. Psychiatry Res (2012) 198:116–22. 10.1016/j.psychres.2011.10.007 22377577

[7] Pitel AL Beaunieux H Witkowski T Vabret F de la Sayette V Viader F Episodic and working memory deficits in alcoholic Korsakoff patients: the continuity theory revisited. Alcohol Clin Exp Res (2008) 32:1229–41. 10.1111/j.1530-0277.2008.00677.x 18482159

[8] Quaglino V De Wever E Maurage P Relations between cognitive abilities, drinking characteristics, and emotional recognition in alcohol dependence: a preliminary exploration. Alcohol Clin Exp Res (2015) 39:2032–8. 10.1111/acer.12841 26332272

[9] Beatty WW Hames KA Blanco CR Nixon SJ Tivis LJ. Visuospatial perception, construction and memory in alcoholism. J Stud Alcohol (1996) 57:136–43. 10.15288/jsa.1996.57.136 8683962

[10] Munro CA Saxton J Butters MA. The neuropsychological consequences of abstinence among older alcoholics: a cross-sectional study. Alcohol Clin Exp Res (2000) 24:1510–6. 10.1111/j.1530-0277.2000.tb04569.x 11045859

[11] Sullivan EV Rosenbloom MJ Pfefferbaum A. Pattern of motor and cognitive deficits in detoxified alcoholic men. Alcohol Clin Exp Res (2000) 24:611–21. 10.1111/j.1530-0277.2000.tb02032.x 10832902

[12] Ambrose ML Bowden SC Whelan G. Thiamin treatment and working memory function of alcohol-dependent people: preliminary findings. Alcohol Clin Exp Res (2001) 25:112–6. 10.1111/j.1530-0277.2001.tb02134.x 11198705

[13] Brandt J Butters N Ryan C Bayog R. Cognitive loss and recovery in long-term alcohol abusers. Arch Gen Psychiatry (1983) 40:435–42. 10.1001/archpsyc.1983.01790040089012 6838323

[14] Fein G Bachman L Fisher S Davenport L. Cognitive impairments in abstinent alcoholics. West J Med (1990) 152:531–7. 2190421 PMC1002406

[15] Oscar-Berman M Kirkley SM Gansler DA Couture A. Comparisons of Korsakoff and non-Korsakoff alcoholics on neuropsychological tests of prefrontal brain functioning. Alcohol Clin Exp Res (2004) 28:667–75. 10.1097/01.ALC.0000122761.09179.B9 15100620 PMC4074361

[16] Ratti MT Soragna D Sibilla L Giardini A Albergati A Savoldi F Cognitive impairment and cerebral atrophy in “heavy drinkers”. Prog Neuropsychopharmacol Biol Psychiatry (1999) 23:243–58. 10.1016/S0278-5846(98)00103-1 10368867

[17] Zhang XL Begleiter H Projesz B. Is working memory intact in alcoholics? An ERP study. Psychiatry Res (1997) 75:75–89. 10.1016/S0925-4927(97)00043-7 9351490

[18] Smith ME Oscar-Berman M. Resource-limited information processing in alcoholism. J Stud Alcohol (1992) 53:514–8. 10.15288/jsa.1992.53.514 1405646

[19] Joyce EM Robbins TW. Frontal lobe function in Korsakoff and non-Korsakoff alcoholics: planning and spatial working memory. Neuropsychologia (1991) 29:709–23. 10.1016/0028-3932(91)90067-I 1944873

[20] Bechara A Dolan S Denburg N Hindes A Anderson SW Nathan PE. Decision-making deficits, linked to a dysfunctional ventromedial prefrontal cortex, revealed in alcohol and stimulant abusers. Neuropsychologia (2001) 39:376–89. 10.1016/S0028-3932(00)00136-6 11164876

[21] Glenn SW Errico AL Parsons OA King AC Nixon SJ. The role of antisocial, affective, and childhood behavioral characteristics in alcoholics’ neuropsychological performance. Alcohol Clin Exp Res (1993) 17:162–9. 10.1111/j.1530-0277.1993.tb00742.x 8452198

[22] Beatty WW Katzung VM Nixon SJ Moreland VJ. Problem-solving deficits in alcoholics: evidence from the California Card Sorting Test. J Stud Alcohol (1993) 54:687–92. 10.15288/jsa.1993.54.687 8271804

[23] Noel X Paternot J Van der Linden M Sferrazza R Verhas M Hanak C Correlation between inhibition, working memory and delimited frontal area blood flow measure by 99mTc-Bicisate SPECT in alcohol-dependent patients. Alcohol Alcohol (2001) 36:556–63. 10.1093/alcalc/36.6.556 11704622

[24] Ratti MT Bo P Giardini A Soragna D. Chronic alcoholism and the frontal lobe: which executive functions are imparied? Acta Neurol Scand (2002) 105:276–81. 10.1034/j.1600-0404.2002.0o315.x 11939939

[25] Zinn S Stein R Swartzwelder HS. Executive functioning early in abstinence from alcohol. Alcohol Clin Exp Res (2004) 28:1338–46. 10.1097/01.ALC.0000139814.81811.62 15365304

[26] Chanraud S Martelli C Delain F Kostogianni N Douaud G Aubin HJ Brain morphometry and cognitive performance in detoxified alcohol-dependents with preserved psychosocial functioning. Neuropsychopharmacology (2007) 32:429–38. 10.1038/sj.npp.1301219 17047671

[27] Donovan DM Kivlahan DR Walker RD. Clinical limitations of neuropsychological testing in predicting treatment outcome among alcoholics. Alcohol Clin Exp Res (1984) 8:470–5. 10.1111/j.1530-0277.1984.tb05704.x 6391258

[28] Walker RD Donovan DM Kivlahan DR O’Leary MR Length of stay, neuropsychological performance, and aftercare: influences on alcohol treatment outcome. J Consult Clin Psychol (1983) 51:900–11. 10.1037/0022-006X.51.6.900 6317722

[29] Fein G Torres J Price LJ Di Sclafani V. Cognitive performance in long-term abstinent alcoholic individuals. Alcohol Clin Exp Res (2006) 30:1538–44. 10.1111/j.1530-0277.2006.00185.x 16930216 PMC1868685

[30] Buckman JF Bates ME Cisler RA. Social networks and their influence on drinking behaviors: differences related to cognitive impairment in clients receiving alcoholism treatment. J Stud Alcohol Drugs (2007) 68:738–47. 10.15288/jsad.2007.68.738 17690808 PMC2947198

[31] Buckman JF Bates ME Morgenstern J. Social support and cognitive impairment in clients receiving treatment for alcohol- and drug-use disorders: a replication study. J Stud Alcohol Drugs (2008) 69:738–46. 10.15288/jsad.2008.69.738 18781249 PMC2575395

[32] American Psychological Association. Publication Manual of the American Psychological Association. Washington, DC: American Psychological Association (1994).

[33] Duka T Townshend JM Collier K Stephens DN. Impairment in cognitive functions after multiple detoxifications in alcoholic inpatients. Alcohol Clin Exp Res (2003) 27:1563–72. 10.1097/01.ALC.0000090142.11260.D7 14574226

[34] Reinert DF Allen JP. The Alcohol Use Disorders Identification Test (AUDIT): a review of recent research. Alcohol Clin Exp Res (2002) 26:272–9. 10.1111/j.1530-0277.2002.tb02534.x 11964568

[35] Weissman MM Bothwell S. Assessment of social adjustment by patient self-report. Arch Gen Psychiatry (1976) 33:1111–5. 10.1001/archpsyc.1976.01770090101010 962494

[36] Heatherton TF Kozlowski LT Frecker RC Fagerstrom KO The Fagerstrom test for nicotine dependence: a revision of the Fagerstrom Tolerance Questionnaire. Br J Addict (1991) 86:1119–27. 10.1111/j.1360-0443.1991.tb01879.x 1932883

[37] Vabret F Lannuzel C Cabe N Ritz L Boudehent C Eustache F [Alcohol-related neuropsychological impairments: nature, impact and detection]. Presse Med (2016) 45:1124–32. 10.1016/j.lpm.2016.01.030 27039333

[38] Noel X Billieux J Van der Linden M Dan B Hanak C de Bournonville S Impaired inhibition of proactive interference in abstinent individuals with alcoholism. J Clin Exp Neuropsychol (2009) 31:57–64. 10.1080/13803390801982726 18608675

[39] Noel X Van der Linden M Schmidt N Sferrazza R Hanak C Le Bon O Supervisory attentional system in nonamnesic alcoholic men. Arch Gen Psychiatry (2001) 58:1152–8. 10.1001/archpsyc.58.12.1152 11735844

[40] Pitel AL Beaunieux H Witkowski T Vabret F Guillery-Girard B Quinette P Genuine episodic memory deficits and executive dysfunctions in alcoholic subjects early in abstinence. Alcohol Clin Exp Res (2007) 31:1169–78. 10.1111/j.1530-0277.2007.00418.x 17511749 PMC2895973

[41] Fama R Pfefferbaum A Sullivan EV. Perceptual learning in detoxified alcoholic men: contributions from explicit memory, executive function, and age. Alcohol Clin Exp Res (2004) 28:1657–65. 10.1097/01.ALC.0000145690.48510.DA 15547452

[42] Beatty WW Katzung VM Moreland VJ Nixon SJ. Neuropsychological performance of recently abstinent alcoholics and cocaine abusers. Drug Alcohol Depend (1995) 37:247–53. 10.1016/0376-8716(94)01072-S 7796719

[43] Goldstein RZ Leskovjan AC Hoff AL Hitzemann R Bashan F Khalsa SS Severity of neuropsychological impairment in cocaine and alcohol addiction: association with metabolism in the prefrontal cortex. Neuropsychologia (2004) 42:1447–58. 10.1016/j.neuropsychologia.2004.04.002 15246283

[44] Pitel AL Witkowski T Vabret F Guillery-Girard B Desgranges B Eustache F Effect of episodic and working memory impairments on semantic and cognitive procedural learning at alcohol treatment entry. Alcohol Clin Exp Res (2007) 31:238–48. 10.1111/j.1530-0277.2006.00301.x 17250615

[45] Moscovitch M Melo B. Strategic retrieval and the frontal lobes: evidence from confabulation and amnesia. Neuropsychologia (1997) 35:1017–34. 10.1016/S0028-3932(97)00028-6 9226662

[46] Weingartner HJ Andreason PJ Hommer DW Sirocco KY Rio DE Ruttimann UE Monitoring the source of memory in detoxified alcoholics. Biol Psychiatry (1996) 40:43–53. 10.1016/0006-3223(95)00290-1 8780854

[47] Baddeley AD Logie RH Working Memory. Oxford: Oxford University Press (1986).

[48] Errico AL King AC Lovallo WR Parsons OA. Cortisol dysregulation and cognitive impairment in abstinent male alcoholics. Alcohol Clin Exp Res (2002) 26:1198–204. 10.1111/j.1530-0277.2002.tb02656.x 12198394

[49] Glenn SW Parsons OA Sinha R Stevens L. The effects of repeated withdrawals from alcohol on the memory of male and female alcoholics. Alcohol Alcohol (1988) 23:337–42. 10.1093/oxfordjournals.alcalc.a044826 3228455

[50] Ceballos NA. Tobacco use, alcohol dependence, and cognitive performance. J Gen Psychol (2006) 133:375–88. 10.3200/GENP.133.4.375-388 17128957

[51] Reitz C den Heijer T van Duijn C Hofman A Breteler MM. Relation between smoking and risk of dementia and Alzheimer disease: the Rotterdam study. Neurology (2007) 69:998–1005. 10.1212/01.wnl.0000271395.29695.9a 17785668

[52] Zhong G Wang Y Zhang Y Guo JJ Zhao Y. Smoking is associated with an increased risk of dementia: a meta-analysis of prospective cohort studies with investigation of potential effect modifiers. PLoS One (2015) 10:e0118333. 10.1371/journal.pone.0118333 25763939 PMC4357455

