# Supplementary material for: Neuropsychological Impairment in Detoxified Alcohol-Dependent Subjects with Preserved Psychosocial Functioning
Source: Front Psychiatry. 2017 Sep 29;8:193. doi: 10.3389/fpsyt.2017.00193 (PMC5626858; doi:10.3389/fpsyt.2017.00193)
Supplement: Supplementary file 1 [file Presentation_1.PDF]

APPENDIX

1 - Mini Mental State (MMSE) (Folstein et al., 1975): The test globally assesses spatio-temporal orientation, learning skills and memory, construction ability, attention, and calculation. It consists of 13 questions administered directly to the subject. The range of possible scores is 0 to 30 where 0 indicates severe impairment and 30 no impairment.

2 - Rey-Osterrieth Complex Figure (ROCF) (Osterrieth, 1944): This test evaluates visuospatial construction, planification and visual memory. First, the subject is asked to copy a complex figure. Then, after a 3-minutes interferent task, the subject is asked to reproduce the figure from memory.

3 - Letter Fluency Test (Cardebat et al., 1990) : This test evaluates executive functioning, such as spontaneous mental flexibility. The subject is asked to generate orally as many words as possible, in two minutes, that begin with particular letters R then P (excluding proper names). This test also assesses the lexical stock when the subject is asked to generate words according to specific semantic categories (e.g. : animals). Performance is measured by the total number of correct words produced.

4 - Trail-Making Test, Parts A and B (TMT A/B) (Reitan, 1956) :

4.1 : TMT A: TMT A evaluates visuomotor and attentional processing (Moriyama et al., 2002). The subject is requested to draw lines to connect 25 consecutively numbered circles, which were randomly distributed on the screen. The time taken for completion was computed.

4.2 : TMT B: This test requires executive functioning with reactive mental flexibility, and divided attention (Moriyama et al., 2002). The subject is requested to draw lines to connect 25 consecutively numbered and lettered circles by alternating between the two sequences. The time taken for completion was calculated.

5 - Stroop Test (Treisman and Fearnley, 1969): This test assesses selective attention and executive functions : inhibitory control and cognitive flexibility. This test is comprised of three separate tasks. During each task, a separate sheet with 100 stimuli is presented to the subject. In the first (word reading), the subject is asked to read aloud the names of colors. In the second (Color naming), the subject is asked to name the ink colors aloud. In the third (color word naming), the word and the ink color are different, the subject is asked to state the ink colors. Comparison between results from the first and third tasks gives an interference score to evaluate subjects' capacities.

6 - Wisconsin Card Sorting Test (WCST) (Heaton and Pendleton, 1981):

The WCST evaluates executive functions, such as : criteria generation and rule maintaining. During this test : four cards with different symbols are placed in front of the subject who is asked to choose one criteria (color, shape, number) to match each card. After each card is placed, the investigator states whether the selected choice is correct. If the choice is correct, the subject continues using the same criteria. If the choice in incorrect, the subject must choose a different criteria. The test proceeds until six sorting categories have been placed. Three scores were calculated: number of categories completed, total errors and perseverative errors.

Subtests of the Weschsler Adult Intelligence Scale III (Wechsler, 1981), WAIS III were:

7 - Information: this subtest assesses long-term memory and more specifically : semantic memory. This subtest is influenced by an individual's level of curiosity, extensiveness of reading and motivation to learn new things, but is independent of subjects' academic level. The subject is asked to answer 28 questions on general knowledge.

8 - Forward and Backward Digit Span Forward digit span evaluates verbal short term memory and attention and backward digit span evaluates verbal working memory. Beginning with a series of two digits in length, each digit string is read aloud. The subject is asked to repeat the digits in either the same order (forward version) or the reverse order (backward version). If either trial of a given length was correctly recalled, then the series length was increased by one, up to a maximum of 9 digits. The task ends when a participant fails both trials of a given length.

9 - Letter-Number Sequencing : This test assesses verbal working memory and the ability to simultaneously recall and organize stimuli.The examiner presented combinations of letters and numbers, from 2 to 9 characters long. The subject is required to repeat each series by, first, repeating the numbers in ascending order, then the letters in alphabetical order (e.g., 9-L-2-A; correct response is 2-9-A-L).

The subtest of the Weschsler Memory Scale III (WMS III)(Wechsler, 1997).

10 - Forward and Backward Spatial span

Forward spatial span evaluates visual short term memory and attention and backward spatial span evaluates visual working memory. This task consists of a board with 10 blocks in fixed locations. The experimenter taps on a series of blocks, then the participant repeats the series either in the same order (forward version) or in the reverse order (backward version). The series gradually increase, with two trials for each series length until the participant is unable to correctly repeat any trials of a given length. Scoring was identical to that of the Forward and Backward Digit Span Task.

11 - Free and cued selective reminding test (FCSRT) (Grober et Buschke., 1988)

This test evaluates verbal episodic memory : Identification, encoding, retention, retrieval and consolidation.

In the FCSRT, encoding is controlled by asking the subject to identify each word to be remembered by pointing and reading it aloud in response to its semantic category. All 16 words have to be retrieved at immediate recall before memory assessment begins. Then, recall is first assessed through free recall, then through cued recall for the missing words. This procedure is repeated three times to give the subject the opportunity to improve performance and provides two main scores: free and total (free + cued) recall. Following the last free and cued recall trials, a yes–no recognition memory for the items is tested by presenting one word at a time. After a delay of 20 minutes during which participants are engaged in other neuropsychological tests a free recall followed by cued recall is proposed. A free delayed recall score and a total delayed recall score (free + cued) are then calculated.
